# Genome-wide association study reveals a QTL and strong candidate genes for umbilical hernia in pigs on SSC14

**DOI:** 10.1186/s12864-018-4812-9

**Published:** 2018-05-29

**Authors:** Eli Grindflek, Marianne H. S. Hansen, Sigbjørn Lien, Maren van Son

**Affiliations:** 1 Norsvin SA, Storhamargata 44, 2317 Hamar, Norway; 2 Department of Animal and Aquacultural Sciences, Centre for Integrative Genetics (CIGENE), Norwegian University of Life Sciences, PO Box 5003, Ås, Norway

**Keywords:** Genome-wide association study, GWAS, Umbilical, Hernia, Pigs, LIF, OSM

## Abstract

**Background:**

Umbilical hernia is one of the most prevalent congenital defect in pigs, causing economic losses and substantial animal welfare problems. Identification and implementation of genomic regions controlling umbilical hernia in breeding is of great interest to reduce incidences of hernia in commercial pig production. The aim of this study was to identify such regions and possibly identify causative variation affecting umbilical hernia in pigs. A case/control material consisting of 739 Norwegian Landrace pigs was collected and applied in a GWAS study with a genome-wide distributed panel of 60 K SNPs. Additionally candidate genes were sequenced to detect additional polymorphisms that were used for single SNP and haplotype association analyses in 453 of the pigs.

**Results:**

The GWAS in this report detected a highly significant region affecting umbilical hernia around 50 Mb on SSC14 (*P* < 0.0001) explaining up to 8.6% of the phenotypic variance of the trait. The region is rather broad and includes 62 significant SNPs in high linkage disequilibrium with each other. Targeted sequencing of candidate genes within the region revealed polymorphisms within the Leukemia inhibitory factor (*LIF*) and Oncostatin M (*OSM*) that were significantly associated with umbilical hernia (*P* < 0.001).

**Conclusions:**

A highly significant QTL for umbilical hernia in Norwegian Landrace pigs was detected around 50 Mb on SSC14. Resequencing of candidate genes within the region revealed SNPs within *LIF* and *OSM* highly associated with the trait. However, *because of* extended LD within the region, studies in other populations and functional studies are needed to determine whether these variants are causal or not. Still without this knowledge, SNPs within the region can be used as genetic markers to reduce incidences of umbilical hernia in Norwegian Landrace pigs.

## Background

Hernias are of the most common congenital defects in pigs which often leads to poor animal welfare and severe economic losses for pig producers. The most common types of hernias in pigs are umbilical and inguinal/scrotal hernia. Umbilical hernia is diagnosed by the protrusion of abdominal contents beneath the skin at the navel (umbilicus). It is generally accepted that weakened supportive muscles around the umbilical stump or navel area of the animal causes the umbilical opening not to close properly and intestines protrude through the intestinal wall to form the “ball-like” structure. The threat of a hernia is the potential entrapment of intestines through this opening. Physical injury, nutrition, excessive pressure, muscular weakness and heredity have been offered as causes of hernia [1]. Umbilical hernias often appears in pigs at 9 to 14 weeks of age, with incidences reported to range from 0.4 to 1.2% [1, 2]. The prevalence of scrotal/inguinal hernias is shown to be approximately in the same range, between 0.5 and 1.5%, and with an estimated heritability of around 0.3 in different breeds [3, 4]. Several studies have explored the genomics of scrotal/inguinal hernia [4–7], but very few have devoted efforts to decipher the genetic architecture of umbilical hernia. This is mainly because umbilical hernia is relatively rare and sometimes complicated to diagnose properly.

It is generally accepted that genetics influence congenital umbilical hernias, but mode of inheritance and genetic architecture are more or less undiscovered, and h^2^ estimates of umbilical hernia are reported to be very low in pigs (0.06–0.08) [1, 8]. The frequencies and heritabilities of umbilical hernia also differs between species. In cattle, for example, both frequencies (0.1–15%) and heritability (h^2^ = 0.4) are reported to be much higher [9, 10] than in pigs. A few studies have tried to decipher the genetic structure of the umbilical hernia. Ding et al. [7] observed significant linkage between markers and umbilical hernia in pigs on 12 different chromosomes, while Ron et al. [11] detected a locus on chromosome 8 linked with umbilical hernia in cattle. Recently, Long et al. [12] published a study suggesting a copy number variation (CNV) on SSC14 to be involved in development of umbilical hernia in pig. Also in humans some studies have been done on structural variations like deletions and duplications, suggesting that such variations also could play a role in occurrence of umbilical hernia [13–15]. Additionally, two studies in other species suggest that umbilical hernia is associated with the function of cyclin-dependent kinase inhibitory protein p57^KIP2^, a regulator of cell proliferation [16, 17].

Studies on genetic control of umbilical hernia so far is not clear, although indicating that the disorder is complex and affected by multiple causative genes and variants. Therefore, the aim of this study was to collect a proper case/control material and identify genomic regions affecting the frequency of umbilical hernia in pigs, using GWAS on a high resolution SNP panel followed up by candidate gene approaches.

## Methods

### Animals and phenotypes

The overall frequency of umbilical hernia for Landrace in Norwegian nucleus herds was found to be only 0.55%, and the heritability (h^2^) was also estimated to be very low (0.065). Due to the low incidence of umbilical hernia in Norwegian Landrace, samples had to be collected over time-periods of altogether 4 years. To generate sufficient number of case/control samples we collected data and samples from all the nucleus herds in Norsvin (Hamar, Norway). Blood samples were collected from a total of 369 purebred Norwegian Landrace piglets displaying umbilical hernia, along with blood from 202 phenotypically unaffected fullsibs within all affected litters. The affected pigs were distributed on 259 litters, with each litter containing 1 to 6 affected piglets from 110 sires. Altogether, 168 parents were genotyped. Samples were obtained from 35 pig breeding farms, where several sires were used on different farms. The farmers reported the cases and thereafter the diagnostic procedures were performed by a Norsvin-breeding technician. The umbilical hernias were subjectively classified into two categories by approximate size “small” or “large” (approximately smaller or larger than baseball size, respectively). Based on this, 165 hernias were classified as “small” and 267 were classified as “large”. Additionally, the time of first observation was recorded, together with records on whether the piglet previously had a history of navel inflammation or not. The size-score of the umbilical hernias was considered not to be valuable as a covariate in the analysis. The first observation of hernias ranged between 2 and 24 weeks, with the average of 14 weeks. Only 2 piglets were previously observed to have an inflammation, and this was therefore not accounted for in the statistical analysis. All animals were cared for according to laws and internationally recognized guidelines and regulations controlling experiments with live animals in Norway (The Animal Protection Act of December 20th, 1974 (revision: FOR-2010-08-06-117), and the Animal Protection Ordinance Concerning Experiments with Animals of January 15th, 1996), according to the rules given by Norwegian Animal Research Authority.

### DNA extraction

DNA was extracted from porcine blood, leukocytes or semen using the MagAttract DNA Blood Midi M48 protocol on the Bio-Robot M48 (Qiagen, Hilden, Germany). The concentration and quality of samples were measured on a Nanodrop, ND-1000 spectrophotometer (NanoDrop Technologies, DE, USA), and on a 1420 Victor plate reader (Turku, Finland) using PicoGreen fluorescence (Molecular Probes, OR, USA), and thereafter normalized to 50 ng/ul in 96-well plates.

### Genotyping, sequencing and quality control

#### Genome wide association study (GWAS)

The genotyping was performed at CIGENE (www.cigene.no), Norwegian University of Life Sciences, Norway. Genotyping for the GWAS was performed using the iScan (Illumina, San Diego, CA, USA) platform with the PorcineSNP60 array according to manufacturer’s instructions. Image intensity, data processing, clustering and genotype calling were performed using the genotyping module in the Genome Studio software (Illumina, San Diego, CA, USA). Altogether, including the hernia pigs, their full-sibs and their parents, 739 Norwegian Landrace were genotyped for 64,232 SNPs. The SNP markers passing the quality control had call rate above 0.9, minor allele frequency (MAF) above 0.01, proportion of genotyped above 0.25, and proportion of genotype errors below 0.025. The average call rate across samples was 0.997, and no samples were excluded from analysis due to unacceptable call rates from the genotyping. Animals with pedigree errors (altogether 17 animals) were removed from the study. After quality control, 554 animals were available for the case/control, in addition to 168 parents. The most frequent failing category is non-informative markers (12,259 SNPs). Beagle v. 3.3.1 was used to impute sporadically missing genotypes in the final genotype file [18]. In total 49,049 high quality SNP markers, were positioned in the porcine genome assembly Build 10.2 (Sanger Institute).

#### Candidate gene study

Candidate genes were chosen based on GWAS results in this study and putative role in development of umbilical hernia from literature [6, 16, 17, 19–25]. In total, eight positional and/or biological candidate genes in seven different regions were selected for sequencing: *Leukemia inhibitory factor* (*LIF*), *Oncostatin-M* (*OSM*), *Macrophage migration inhibitory factor* (*MIF*), *cyclin-dependent kinase inhibitory protein p57KPI2* (*CDKN1C*), *Pyrroline-5-carboxylate reductase 1* (*PYCR1*), *Versican* (*VCAN*), *Matrix metallopeptidase-13* (*MMP13*), and *Vimentin* (*VIM*). Names, positions from www.ensembl.org and references are listed in Table 1.

**Table 1:** Eight positional and/or biological candidate genes were used for resequencing

Gene name	Position^a^	References
Leukemia inhibitory factor (LIF)	14:50263722–50,266,807	[19]
Oncostatin M (OSM)	14:50281701–50,286,188	[20, 21]
Macrophage migration inhibitory factor (MIF)	14:53282552–53,283,339	[22]
Cyclin-dependent kinase inhibitory protein p57KPI2 (CDKN1C)	Unplaced	[16, 17]
Pyrroline-5-carboxylate reductase 1 (PYCR1)	Unplaced	[6]
Versican (VCAN)	2:93783042–93,895,497	[23]
Matrix metallopeptidase 13 (MMP13)	9:37493636–37,504,552	[24]
Vimentin (VIM)	10:48227005–48,235,670	[25]

^a^Positions are in chromosome:basepairs

SNP discovery was performed by PCR resequencing of genomic DNA and cDNA from Norwegian Landrace with umbilical hernia and healthy siblings. Primers were designed using Primer3 [26]. The programmes Phrap, Phred and PolyPhred (v.4.06) were used to identify putative SNPs from the PCR resequencing chromatograms, and the Consed programme was used to visually confirm the putative SNPs [27]. For the SNP detection, eight hernia pigs and eight healthy pigs were used for sequencing. No SNPs were detected in the positional candidates *MIF* and *CDKN1C*, but 50 SNPs were obtained in six other genes. Primer assays for use in the Sequenom MassARRAY system were designed using MassARRAY Assay Design Software, with multiplexes between 12 and 19. Genotyping was done according to the manufacturer’s instructions in the IPLEX protocol. The MassARRAY Typer software was used for the automated genotype calling (Sequenom, San Diego, USA). Altogether, 46 SNPs in six different candidate genes were successfully genotyped for 463 animals; 201 pigs with umbilical hernia, 135 healthy siblings and 127 parents. Due to high SNP density in *VCAN* (16 SNPs) the six SNPs with highest MAF were selected, and additional five SNPs located in other genes were removed due to low MAF (MAF < 0.01). Therefore, 31 SNPs were used for the final statistical analyses (Table 2). Candidate genes with more than one SNP were included for haplotype analysis. Haplotypes and LD blocks were constructed using the software Haploview v.4.2 [28].

**Table 2:** SNPs within candidate genes used for association analysis

Gene Name	SNP name	Location	Alleles^a^	MAF^b^	P^c^	%var^d^
Versican	VCAN_1	Exon 8	A/**G**	0.45	n.s.	0.2
Versican	VCAN_2	Exon 8	G/**A**	0.45	n.s.	0.4
Versican	VCAN_3	Exon 8	T/**G**	0.45	n.s.	0.4
Versican	VCAN_4	Exon 8	C/**T**	0.45	n.s.	0.2
Versican	VCAN_5	Exon 8	**C**/A	0.38	n.s.	0.4
Versican	VCAN_6	Exon 8	C/**T**	0.38	n.s.	0.4
Leukemia inhibitory factor	LIF_1	Intron 1	**A**/G	0.47	0.001	8.6
Matrix metallopeptidase 13	MMP13_1	Intron 7	**G**/A	0.33	n.s.	0.08
Matrix metallopeptidase 13	MMP13_2	Intron 7	**T**/G	0.33	n.s.	0.05
Matrix metallopeptidase 13	MMP13_3	Exon 8	**A**/G	0.33	n.s.	0.1
Matrix metallopeptidase 13	MMP13_4	Intron 8	**C**/T	0.33	n.s.	0.03
Matrix metallopeptidase 13	MMP13_5	Intron 8	**T**/C	0.32	n.s.	0.01
Matrix metallopeptidase 13	MMP13_6	Intron 8	**G**/T	0.33	n.s.	0.3
Oncostatin M	OSM_1	Exon 3	**C**/T	0.47	0.001	7.7
Oncostatin M	OSM_2	Exon 3	**T**/C	0.47	0.001	8.6
Oncostatin M	OSM_3	Exon 3	**T**/C	0.47	0.001	8.6
Oncostatin M	OSM_4	Exon 3	**G**/T	0.47	0.0008	8.6
Oncostatin M	OSM_5	Exon 3	**A**/G	0.47	0.001	8.6
Oncostatin M	OSM_6	Intron 3	**C**/T	0.47	0.001	7.7
Oncostatin M	OSM_7	Intron 3	**T**/C	0.47	0.001	7.7
Oncostatin M	OSM_8	Intron 3	**A**/G	0.47	0.001	7.7
Pyrroline-5-carboxylate reductase 1	PYCR1_1	5’ UTR	**A**/T	0.11	n.s.	2.5
Pyrroline-5-carboxylate reductase 1	PYCR1_2	5’ UTR	**C**/T	0.13	n.s.	1.0
Pyrroline-5-carboxylate reductase 1	PYCR1_3	Intron 1	**C**/T	0.03	n.s.	1.0
Vimentin	VIM_1	Intron 5	C/**T**	0.21	n.s.	0.2
Vimentin	VIM_2	Intron 5	A/**G**	0.36	n.s.	0.3
Vimentin	VIM_3	Intron 5	C/**T**	0.17	n.s.	0.01
Vimentin	VIM_4	Intron 5	A/**G**	0.19	n.s.	0.6
Vimentin	VIM_5	Intron 5	C/**T**	0.15	n.s.	0.00
Vimentin	VIM_6	Intron 5	C/**A**	0.34	n.s.	0.3
Vimentin	VIM_7	Intron 5	C/**A**	0.11	n.s.	0.7

^a^major/minor allele, where risk allele is indicated in bold

^b^minor allele frequency (MAF)

^c^*p*-value (*p* > 0.001 is n.s)

^d^the percentage of explained phenotypic variance (%var)

Structural models of the OSM protein were generated by using the SWISS-MODEL workspace [29], using amino acid sequence input files for the OSM translated from DNA sequence with the two different alleles of the missense single point mutation. QMEAN [30] were used to evaluate the quality of the model in 6 different terms: (1) C_beta interaction energy, (2) all-atom pairwise energy, (3) solvation energy, (4) torsion angle energy, (5) solvent accessibility agreement and (6) total QMEAN-score. Furthermore, GROMOS empirical force field energy [31] was estimated for each amino acid of the protein chain.

### Statistical analyses

#### Genome wide association study (GWAS)

The association analyses were carried out for the 49,049 SNPs mapped on the 18 porcine autosomes using pig genome assembly Build10.2. The minor allele frequencies of the selected SNPs were uniformly distributed between 0.01 and 0.5. Genome-wide association analysis was performed with the GenABEL package (version 1.6–5) in R [32] using the structured association approach (“qtscore”) for binary traits [33], which is in principle a Cochran-Armitage test. Umbilical hernia was the binomial trait and gender was added as a covariate, although significant differences between gender was not observed (*p* = 0.7). Gender-factor have not either previously been reported in pigs, but in cattle, however, umbilical hernia have been found to be more frequently occurring in female compared to males [10].

The *p*-values corrected for genomic control (GC) of a 1-df test were accepted to represent proof of genome-wide association at *p* < 0.001 (−log10(p) = 3). Collection of samples was done during intensive periods of altogether four years, but it was seven years between the first and final collection of samples. Therefore, a population stratification analysis (“strata”) was performed to find whether the population during some generations of selection was grouped into several genetic populations or not. However, only one population was defined and correction for stratification was not added in the final analysis.

#### Candidate gene study

For the single SNP-marker and haplotype association analysis the ASReml v.2.00 [34] was used to conduct association analysis using the following model:

$$ \mathrm{Y}=\upmu +\mathrm{snp}/\mathrm{haplotype}+\mathrm{sex}+\mathrm{id}+\mathrm{e} $$

where Y is the binomial trait “case” or “control”. The SNP, coded as 0, 1, or 2 for homozygote allele 1, heterozygote, homozygote allele 2, respectively, was fitted as a fixed effect. In case of the haplotype associations, each haplotype allele combination was assigned its own level and fitted as a fixed effect. Sex was fitted as a fixed effect whereas animal id was treated as a random effect, using a pedigree based relationship matrix to account for population structure. The F-statistics was calculated for each SNP or haplotype and the significance level was set at a corresponding *p*-value of 0.001. The genetic variance explained by a SNP was calculated from the estimated genotype effects and the observed genotype frequencies. It was expressed as a percentage of the total phenotypic variance obtained from the model without the genotype effect.

## Results

### Genome-wide association study (GWAS)

**Fig. 1:**
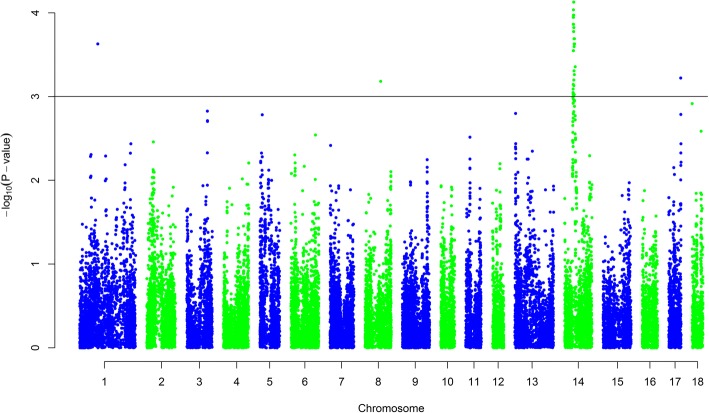
Manhattan plot of genome-wide association results. The x axis represents the genome in physical order whereas the y axis shows –log10 *p*-value for all SNPs. The line corresponds to a significance level of *P* < 0.001

**Fig. 2:**
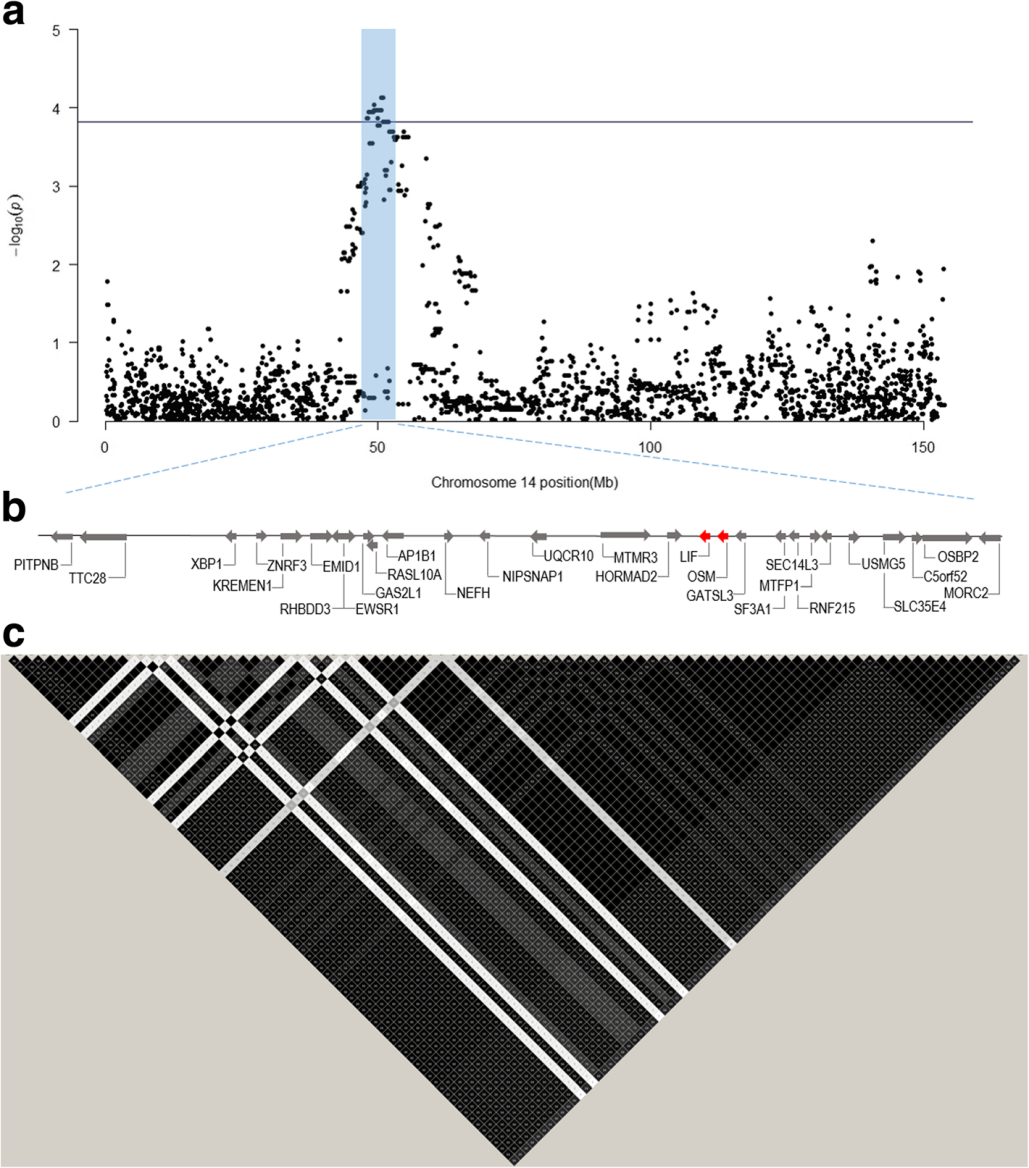
Association results for SSC14. **a** A QTL region of 62 SNPs on SSC 14 reached a significance level of *P* < 0.00015 (solid line) and they were localized between 48.04 and 50.99 Mb. **b** Map of characterized genes in the QTL region and their orientation, based on information available from Ensembl, where candidate genes are indicated in red. **c** An LD plot was constructed using Haploview for the QTL region. R^2^ was used as a measurement of LD, where a darker color represents a higher R^2^ value. The red triangle covers the SNPs in the candidate genes and they are all in complete LD

Assuming a corrected significance level of *P* < 0.001, 129 SNP markers reached the significant level. Almost all of them, 126 SNPs, were located on SSC14 between positions 47.16 Mb and 58.91 Mb. The other three single SNP associations were obtained on SSC1 around 102 Mb at the Porcine Build10.2 genome sequence (http://www.ensembl.org), on SSC8 around 88 Mb, and on SSC17 around 68 Mb, respectively. The Manhattan plot of all chromosomes is shown in Fig. 1. With a more strict significance level of *P* < 0.0001, 62 SNP markers were significant and all of them were located between 48.04–50.99 Mb at SSC14. The most significant SNPs (*p* < 7 × 10–5) are listed in Table 3. Figure 2 shows the association results for SSC14 with characterized genes and LD within the QTL region (*P* < 0.0001).

**Table 3:** The most significant 60 k SNPs for umbilical hernia

Marker name	SSC	Position	MAF^a^	*P*-value^b^	%var^c^
INRA0043998	14	50,652,198	0.48	7.43e-05	8.1
ASGA0063261	14	50,672,335	0.48	7.43e-05	8.1
ASGA0063262	14	50,738,530	0.48	7.43e-05	8.1
ALGA0077446	14	50,761,608	0.48	7.43e-05	8.1
ASGA0063267	14	50,776,775	0.48	7.43e-05	8.1
ALGA0077450	14	50,792,395	0.48	7.43e-05	8.1
MARC0024032	14	50,824,208	0.48	7.43e-05	8.1
INRA0044003	14	50,847,851	0.48	7.43e-05	8.1
MARC0112737	14	50,917,403	0.48	7.43e-05	8.1
ALGA0077457	14	50,938,144	0.48	7.43e-05	8.1
ASGA0063274	14	50,964,903	0.48	7.43e-05	8.1
H3GA0040130	14	50,992,818	0.50	7.43e-05	7.9
ASGA0063217	14	49,288,281	0.49	9.21e-05	8.6

^a^minor allele frequency (MAF)

^b^the genomic control corrected p-value

^c^the percentage of explained phenotypic variance (%var)

### Candidate genes for umbilical hernia

Six candidate genes were included in the candidate gene association study. Four of them were based on biological knowledge and previously known associations to umbilical hernia, and two of them were chosen based on the QTL position obtained on SSC14 and biological function. The Oncostatin-M precursor gene (*OSM*) and Leukemia inhibitory factor (*LIF*) was the two genes located within the SSC14 QTL. For OSM, all the eight single SNP markers, and the haplotype combining all the eight SNPs, were significantly associated with umbilical hernia (*P* < 0.001) and found to explain up to 8.6% of the phenotypic variance (Tables 2 and 4). As shown in Fig. 2, all eight SNPs within *OSM* are in complete LD with each other. Only two different haplotypes were obtained in the data set, with frequencies of 0.53 and 0.46 for haplotype 1 and 2, respectively. The haplotype frequencies for the affected/un-affected animals were 0.72/0.28 for the 11, 0.54/0.46 for the 12 and 0.55/0.45 for the 22, respectively. Constructing haplotypes within a block between 48 and 51 Mb, covering both *OSM* and *LIF* (see Fig. 2), we got two major haplotypes (0.54 and 0.33) and with remaining haplotypes having a frequency of 0.07 or less. For the homozygote haplotype 1, 80% of the animals had umbilical hernia, for the heterozygote 2 animals 53% had hernia, and for the homozygote 12 51% had hernia, which may suggest a dominant effect of the haplotype, with the haplotype 1 as the risk allele. The OSM_1 SNP located in exon 3 (Table 2) is non-synonymous causing an amino acid shift from Serine to Glycine. This seems to cause only minor changes in the protein structure obtained by SWISS-MODEL [29]. However, the GROMOS empirical force field energy was shown to be positive (unfavorable environment) with Serine and slightly negative (favorable environment) when changed to Glycine (results not shown). Only one polymorphism was obtained by sequencing for the other positional candidate gene (*LIF*) on SCC14. This marker was also significantly associated with umbilical hernia (*p* < 0.001) and found to be in perfect LD with *OSM* (Fig. 2). None of the polymorphisms detected in the four candidate genes outside the QTL-region on SSC14 (*VCAN*, *MMP13*, *PYCR1* and *VIM*) obtained significant associations with umbilical hernia (Table 2).

**Table 4:** Results from the haplotype analyses

Gene name	#haplotypes^a^	*P*-value	% Var^b^
Versican (VCAN)	7	0.03	0.1
Matrix metallopeptidase 13 (MMP13)	5	0.04	0.02
Oncostatin M (OSM)	2	0.0012	6.0
Pyrroline-5-carboxylate reductase 1 (PYCR1)	6	0.03	0.07
Vimentin (VIM)	11	0.13	0.1

^a^haplotypes were constructed within genes based on SNPs in Table 2 and are presented with the number of haplotypes with a frequency > 2%

^b^the percentage of explained phenotypic variance

## Discussion

In this study, a genome wide association analysis was conducted in the Norwegian Landrace population, together with studies of positional and biological candidate genes for umbilical hernia.

The most convincing result from the GWAS was around 50 Mb on SSC14. Three candidate genes in this region were investigated in more detail; *OSM*, *LIF* and *MIF* (Macrophage migration inhibitory factor). *OSM* is located at SSC14 base pair position 50,281,701–50,286,188, *LIF* is located at SSC14 base pair position 50,263,722–50,266,807 and *MIF* at position 53,282,553–53,283,313. The three genes were sequenced at the genomic level in eight hernia pigs and eight healthy pigs, but SNPs were only detected in the *OSM* and *LIF* genes. *OSM* and *LIF* have similar gene structure and functions, both are pleiotropic cytokines that belong to the interleukin-6 (IL-6) family, and they can interact with each other [35]. These cytokines play a crucial role in diverse biological events like growth promotion and cell differentiation, as well as embryogenesis and inflammatory responses to injury and infection [19–21]. *LIF* is shown to be the inducer of the acute phase protein synthesis affecting the cell recruitment into the area of damage or inflammation (reviewed by [19]). MIF is also involved in immune responses by regulation of cytokine secretion and the expression of receptors that are involved in innate immunity [22], but no variations within the *MIF* gene were detected in the pigs resequences in this study. Even though inflammation at the umbilicus at weaning was not previously found to be associated with hernia development [1], our results may suggest the involvement of immunological factors in the development of umbilical hernia in pigs.

*PYCR1* was added as a biological candidate gene due to its importance in conversion of glutamate to proline and suggested role in cell growth regulation [36]. Proline is essential for the stabilization and a major component of collagen, which accounts for three-quarters of the dry weight of skin and is the most prevalent component of the extracellular matrix [37]. Diaphragmatic, inguinal, and umbilical hernia are all associated with connective tissue weakness, and previously extracellular matrix proteins such as collagens, fibronectin, elastin and matrix metalloproteinases have been suggested to be involved [38, 39]. Moreover, a candidate gene study for scrotal hernia in pigs obtained a highly significant association between *PYCR1* and hernia [6]. Several matrix metalloproteinase genes are suggested to be associated with membrane weakening and rupture. One of them, *MMP13* on SSC9, is shown to obtain an important role in wound healing by coordinating cellular activities important in the growth and maturation of granulation tissue, including myofibroblast function, inflammation, angiogenesis, and proteolysis [24]. MMP13 was for example shown to affect the structural integrity and mechanical stability of the connective tissue in both indirect and direct scrotal hernias [38]. The VCAN is shown to display high adhesive ability to endothelial cells and facilitated tube-like structure formation, and is previously suggested as a candidate to be involved in development of umbilical hernia [23]. In this study the *VCAN* gene, located on SSC2, was chosen as a biological candidate gene. Finally, *vimentin* (*VIM*), which is located on SSC10, was added as a biological candidate gene due to its function of being a marker of immature smooth muscle cells and being involved in collagen structure [25], as well as having suggested involvement in inflammatory/immune response [40].

Since there are variability in level of expression incidences of umbilical hernia, we are aware that defining the trait as a case or control trait is one of the limitations in this study. However, no other way of recording the trait have successfully been applied, in either this study or others. Statistical packages suitable for analyzing binary traits were therefore performed for GWAS study as well as for the single SNP/haplotype association study. Limited number of genomic studies have investigated associations between genes/genetic markers and umbilical hernia. A genome wide scan in White Duroc and Erhualian, using 194 microsatellites and two different statistical methods, obtained significant QTL regions for umbilical hernia in altogether 11 chromosomes [7]. The most promising loci were revealed on SSC7 and SSC10. None of the SNPs from our GWAS or candidate study are located within or close to the significant chromosomal regions in Ding et al. [7]. The putative QTLs detected on SSC14 in Ding et al. [7] are located more telomeric (~ 140 and 149 Mb). Also, in a study by Liao et al. [41] two suggestive loci were found on two other locations, SSC2 and SSC17. In cattle, Ron et al. [11] suggested that a umbilical hernia allele found in the study was dominant or codominant with partial penetrance. The locus detected was located on the centromeric end of the bovine chromosome 8, comparative to SSC14 position 14–15 Mb. In our study no significant SNP associations were obtained in, or close to, this region. The discrepancy between studies are probably due to several reasons. Low heritability indicate that it is many genes involved and that the trait is highly influenced by environmental factors. Different causative alleles and a variety of allele frequencies in different breeds would highly affect which QTLs that are detectable in different breeds.

## Conclusions

A highly significant QTL for umbilical hernia in Norwegian Landrace pigs was detected between 48 and 51 Mb on SSC14 (*P* < 0.00015) explaining up to 8.6% of the phenotypic variance for umbilical hernia. Resequencing of candidate genes provided SNPs and haplotypes for association analyses and SNPs within positional candidate genes *LIF* and *OSM* in the SSC14. Due to the large degree of LD in the region, future functional studies are needed to assign the causal variant(s) and further studies should be conducted to investigate the effect of this QTL region in other pig breeds. SNPs within the QTL region can be used as genetic markers to reduce incidences of umbilical hernia in Norwegian Landrace pigs.

## Abbreviations

CDKN1C: Cyclin-dependent kinase inhibitory protein p57KPI2
LIF: Leukemia inhibitory factor
MIF: Macrophage migration inhibitory factor
MMP13: Matrix metallopeptidase-13
OSM: Oncostatin M
PYCR1: Pyrroline-5-carboxylate reductase 1
VCAN: Versican
VIM: Vimentin
